# Hygiene performance rating at farm level - an auditing scheme for evaluation of biosecurity measures’ effect on prevalence of *Campylobacter* from selected broiler producers

**DOI:** 10.1186/s13028-024-00762-w

**Published:** 2024-08-13

**Authors:** Gunvor Elise Nagel-Alne, Ole-Johan Røtterud, Thorbjørn Refsum, Janne Holthe, Miriam Garner, Eystein Skjerve, Sigrun J. Hauge

**Affiliations:** 1 Animalia Norwegian Meat and Poultry Research Center, P.O. Box 396 Økern, Oslo, 05413 Norway; 2 Norsk Kylling, Havneveien 43, Orkanger, 7300 Norway; 3 Faculty of Veterinary Medicine, NMBU Norwegian University of Life Sciences, Ås, 1430 Norway

**Keywords:** Hygiene performance, Poultry, Preventive, Rating protocol, Tool

## Abstract

**Background:**

Preventing pathogens from entering the broiler premises is the main biosecurity measure at farm level. In conventional broiler production, chickens are kept indoors during the entire production period. Pathogens can enter the broiler-producing unit from sources such as water, equipment, personnel, insects, and rodents. The possible routes must be controlled, and corrective measures applied when necessary. The objective of this study was to (1) develop a hygiene protocol and test the scheme on 30 farms, and (2) compare the results to their *Campylobacter*-colonised status. A Hygiene Performance Rating protocol at farm level (HPR-F) was developed to systematically review the production to identify risk areas to biosecurity. The HPR-F consists of 13 categories with related questions. For each question, a score was given from 1 to 3, where 1 meant “acceptable”, 2 was “potential for improvements”, and 3 was “not acceptable”. Scores for each question were multiplied with weight factors for hygienic impact and economic consequences describing whether the necessary improvement depends on a significant investment or is a cheap quick-fix and calculated into a percentage where 100% is perfect hygiene. The 30 farms in the study were selected from one county in Norway. The *Campylobacter-*results for each of the 30 farms in 2019–2021 were given according to rules in the Norwegian Action Plan against *Campylobacter* faecal sampling on-farm 3–6 days prior to slaughter.

**Results:**

The overall results from the HPR-F showed that the general hygiene level was high in all farms. The mean total hygiene score was 82% and varied from 70 to 92%. The category Handling dead chicken had the highest hygiene score (93%), and Ventilation had the lowest score (55%). The HPR-F results were compared to the *Campylobacter*-status for the 30 farms: *Campylobacter*-negative flocks had slightly higher total scores than *Campylobacter*-positive flocks (*P* = 0.19). Among others, the category Outdoor area (vegetation close to the premises’ walls) was identified as the most stable factor in relation to be colonised with *Campylobacter*.

**Conclusions:**

The HPR-F tested in this research trial provides a tool for veterinarians, advisors, and poultry farmers to improve biosecurity at farm level and enhance the preventive animal health initiatives.

## Background

In conventional poultry production, chickens are kept indoors during the entire rearing period, for optimal environmental conditions and to prevent pathogens from entering the flock. However, pathogens can enter via air, water supplies, equipment used outside the premises, feed, personnel, insects, and rodents. All these possible routes for entrance of pathogens into premises should be controlled and corrective measures applied when necessary. Biosecurity, defined as all measures taken to prevent both the introduction and the spread of infectious agents on the farm [1], is of key importance in the prevention of animal disease in general. Despite the knowledge and understanding of the importance of biosecurity in animal production, large variation in the level of performed biosecurity at farms is observed [2].

The most common causes of foodborne zoonoses in Europe, *Campylobacter* and *Salmonella*, are associated with poultry meat [3]. In Norway, *Campylobacter*-colonisation in broiler flocks occurs mostly during the summer period from May to September. A National Action Plan against *Campylobacter* [4] monitors broiler flocks sent to slaughter at < 50 days of age, by sampling faecal material on-farm. For the last five years (2019–2023), the *Campylobacter*-colonisation flock prevalence was 4.8–6.1% [5]. Te flocks in this study were slaughtered at the age of 46 days, and the sampling of faecal material for *Campylobacter-*analysis was performed when the chickens were 40–43 days old. Broiler carcasses from *Campylobacter*-colonized flocks according to the result from faecal sampling on-farm, are either deep frozen (− 18 °C) for more than 3 weeks or heat treated, before being sent to the market [5]. This is an expensive intervention; therefore, it is important to minimise the number of *Campylobacter*-colonised flocks as much as possible. Broiler farms with colonised flocks receive guidance on biosecurity measures.

Several studies have identified risk factors for *Campylobacter* and other pathogens in living poultry. Partial depopulation (thinning) is a risk factor and can be avoided by an “all in-all out” system [6]. In the Cam-con project [7], risk factors were identified for Danish and Norwegian broiler flocks. Risk factors included broiler houses older than five years, longer downtime, broiler-houses without a separate anteroom-barrier, and the use of drinking nipples with cups compared with nipples without cups. A high risk of transfer of *Campylobacter* from flies to broilers during summer season has been described [8]. Other studies have reported that the most effective measure was to build new production facilities with strict biosecurity and again that drinking nipples should be without cups below [9]. Strict handling of manure without contamination to environment and disposed away from the chicken house, showed a lower prevalence of *Campylobacter*-colonisation [10], while rain and daily mean temperatures above 6 °C and private water supply increased the probability for a broiler flock to test positive for *Campylobacter* spp. [11]. Use of insect nets in ventilation systems to prevent insects from entering buildings has also been investigated, and a Danish study reported a reduction in *Campylobacter*-prevalence from 51 to 15% [12]. Part of the reduction can probably also be explained by more focus on management and biosecurity in general.

An initiative on screening biosecurity levels in broiler production is the Biocheck.UGent^®^ scoring system, developed to measure and quantify the level of biosecurity on broiler farms [13]. Another study evaluated the compliance with biosecurity rules in Danish broiler flocks according to a predefined quality system was [14]. Risk factors for *Campylobacter*-colonization of broilers in selected European countries has also been presented [9]. The mentioned systems are all targeted at identifying areas that could hamper biosecurity in general and increase the risk of *Campylobacter*-colonisation in chicken flocks.

This study presents a comprehensive protocol (audit scheme), the Hygiene Performance Rating-Farm (HPR-F), targeted at identifying practices that could hamper the biosecurity level in conventional broiler production. All previously known and identified risk factors were included in the development of the HPR-F audit scheme. A case-control study was established where broiler producers with *Campylobacter*-colonised flocks were compared to producers having no *Campylobacter*-colonised flocks during a period of three years (2019–2021). The aims of this study were (1) to develop the HPR-F protocol and test it on a selected number of farms, and (2) to compare the HPR-F results to the broiler farms’ *Campylobacter*-status to identify risk factors for *Campylobacter*-colonisations in broiler flocks.

## Materials and methods

### Development of the HPR-F protocol

The HPR-F protocol was developed by Animalia in 2019 and addresses factors with impact on hygienic performance and biosecurity of broiler production at farm-level. The construction of HPR-F is based on the same principles as for the slaughter hygiene protocol developed earlier [15].

The factors were divided into 13 categories, namely Structure of the farm, Premises, Outdoor area, Anteroom barrier, Drinking water, Feed and dry litter, Human movement, Manure management, Cleaning routines, Pest control, Ventilation, Carcass handling and Receiving day-old chickens.

A total of 170 questions were distributed in the 13 categories, to address factors being detrimental to biosecurity. Each category consists of three (Carcass handling) to 34 questions (Anteroom barrier). The complete list of questions can be provided on request to the first author. The questions included in HPR-F were based on a thorough study of present literature regarding disease transmission in poultry, general knowledge of biosecurity at farm level, results from a former questionnaire to broiler producers and the formal requirements to be fulfilled by the broiler farmers [16]. Examples of questions in the protocol are whether all air is taken in through intended air intakes and which disinfectants are used and how often.

The questions were answered either through observation by trained assessors or, if not possible, by interviewing the farmers, depending on the nature of the question. The questions were answered by predefined scores from 1 to 3, where 1 meant “acceptable”, 2 was “potential for improvement”, and 3 was “not acceptable”. The score for each question or control point was multiplied by a weight factor for hygienic impact and risk (1, 3, 6 or 12) and economic consequences (1 or 2) according to previous developed system [15]. The weighing factor of each question was established based on the importance of each question from an economic and hygienic standpoint. The assessed hygiene rating according to the farm’s total score, is shown in Table 1 and is based on previously published assessment [15]. The same assessment rating of hygiene is also applied to the individual 13 categories of biosecurity.

**Table 1:** Assessment of hygiene in the hygiene performance rating-farm (HPR-F) scheme

Total score per farm	Assessment of hygiene
85.1–100.0%	Excellent hygiene
70.1–85.0%	Good hygiene
55.1–70.0%	Acceptable hygiene
0–55.0%	Poor hygiene

The HPR-F draft was presented to six assessors, and then revised and improved. The revised protocol was then tested on four farms using several assessors. Minor corrections were conducted after testing.

### Selected farms, study period and data sampling

In total, 30 conventional broiler houses in Trøndelag county in Mid-Norway were included in the study. All 30 farms reared the broiler hybrid Hubbard JA787. One farm had two broiler houses and was visited twice; hence the dataset includes registrations from 30 broiler houses from 29 farms. All farms had an “all in-all out” system with no partial depopulation of broiler flocks.

Farms were visited by one assessor at a time, and a total of six assessors conducted the assessments during 2021 and 2022. The first visit was done with at least one other assessor, to ensure that the assessment of scores was calibrated. Registration of the 170 questions was conducted in an Excel spreadsheet on a tablet and collected for further analysis.

### Campylobacter-status

The *Campylobacter*-status was based on information from the National Action Plan against *Campylobacter* [17]. Faecal samples were collected on farms 3–6 days prior to slaughter and analysed using PCR. For the period 2019–2021, 13 farms had no positive flocks, 7 farms had 1 positive flock, 4 farms had 2 positive flocks, and 6 farms had 3 positive flocks. The maximum number of positive flocks in a farm over three years was three and farms with one, two or three positive flocks were collapsed to one group. The dichotomous dependant variable was denoted (1,0) as having positive flocks (*n* = 17 farms) or not having positive flocks (*n* = 13 farms) according to *Campylobacter*-cases in the National Action Plan against *Campylobacter* in 2019–2021 [17].

### Statistical analysis

Biosafety variables were described using mean values of the 13 registered categories based on the individual scoring of the 170 questions. The statistical analysis was conducted in STATA/BE 17.0 (Stata Corp, College Station, TX) using logistic regression where the outcome variable was *Campylobacter*-status (0,1), and the explanatory variables were the mean hygiene scores of the 13 categories in the HPR-F protocol. Linearity between the outcome *Campylobacter*-status and the explanatory variables was investigated graphically. The degree of normal distribution of the explanatory variables was regarded acceptable as to use mean values in the regression analyses. A logistic regression model with sum of scores was built to analyse relation between *Campylobacter*-status and hygienic performance in the HPR-F protocol.

## Results

### Biosecurity results in the study population – farms and outdoor areas

Most trial farms (90%) had only one broiler house and 10% had two broiler houses. Some of the farms (31%) had other livestock and 69% had cats or other pets on the farm. The farmers reported that livestock was grazing on pasture close to the broiler house during summer on 31% of the farms. The area around the broiler houses (2 m) was without vegetation and stored objects in 62% of the farms, making it more difficult for insects and pests to enter the house. 86% had solid or concrete cover on the ground in front of doors and gates. There were no feed leaks on the ground. All trial farms (100%) slaughtered all chickens in the flock concurrently (all in - all out), and none practised depopulation or thinning.

### Biosecurity results in the study population – housing characteristics

All broiler houses in this study had conventional in-door production. The category “anteroom barrier” consisted of 34 detailed questions about the sluice area for changing shoes, clothes, and washing hands. In 28% of the broiler houses, floors had damages that were assessed as difficult to clean properly. Gates were present at both ends of 55% of the buildings, so that withdrawal and intake take place at opposite ends. In 23% of the houses, ventilation valves were placed on walls, while 77% had ceiling valves. Only 13% used fly/insect nets on all air intakes. One-third of the farmers said that on very hot days, gates and doors were opened. 69% of broiler houses had gable fans, and 20% of them lacked a cover to prevent insects from entering.

### Biosecurity results in the study population - In-and-out of the broiler house

On half of the farms, there were no requirements to document health for personnel, such as absence of diarrhoea when entering the broiler house. However, 89% demanded 48-hour quarantine for people who had visited, worked with or been in contact with poultry abroad before entering the broiler house.

Sawdust, litter, and environmental enrichment were stored outside in such a way that this potentially could be contaminated by wild birds and pests on 20% of the farms. The use of environmental enrichments, which can pose a hygiene problem in relation to contamination from straw without heat treatment, twigs, peat, chopping stones, etc., were valid for 67% of the farms.

The source of water was from public waterworks in 57% of the farms, while the rest received water from smaller private waterworks or their own sources. Half of the private waterworks did not have any form of purification/disinfection, while the other half had UV disinfection. The sequence of particle filter and UV unit was incorrect in several houses with private water supply. Annual bacterial sampling including *Enterobacteriaceae*,* E. coli* and total bacterial count was performed by 92% of those who had private water supply, and all of these had had satisfactory results according to documentation. Maintenance of the UV system did not occur in several cases (3 of 5 farms).

Nine farms had “excellent hygiene” (with a total score of 85.1–100.0%), 20 farms had a total score assessed as “good hygiene” (70.1–85.0%) and one farm had “acceptable hygiene” (55.1–70.0%) as presented in Table 1.

### Distribution of hygiene score

The data in the 13 categories representing different factors of biosecurity were regarded as sufficiently normally distributed and the results are presented by mean numbers. Overall, the mean total hygiene score was 81.9% with a range between 69.2 and 92.2%. The mean values of the thirteen categories of biosecurity factors and the total hygiene score are presented in Table 2 with minimum and maximum values.

**Table 2:** Mean score of hygiene for the thirteen categories of biosecurity and the overall hygiene score

Categories of biosecurity	Mean value	Level of hygiene	SD	Min	Max
Carcass handling	92.8	****	16.2	57.1	100
Pest control	91.8	****	12.4	57.2	100
Anteroom barrier	88.2	****	6.3	70.9	97.9
Manure handling	87.9	****	11.9	59.2	100
Cleaning	87.4	****	8.8	60.0	98.2
Structure on farm	83.6	****	9.9	58.6	100
Premises	82.7	****	20.2	42.4	100
Receive day-old chickens	79.4	***	14.8	50.0	100
Feed and litter	73.1	***	16.6	40.0	100
Outdoor area	70.1	***	19.1	35.8	100
Drinking water	70.1	**	15.5	40.3	100
Human movement	68.2	**	16.1	30.4	96.9
Ventilation	55.4	**	20.6	11.7	100
Overall hygiene	81.8	***	5.73	69.1	92.2

**** Excellent hygiene, *** Good hygiene, ** Acceptable hygiene according to assessment presented in Table 1

**Fig. 1:**
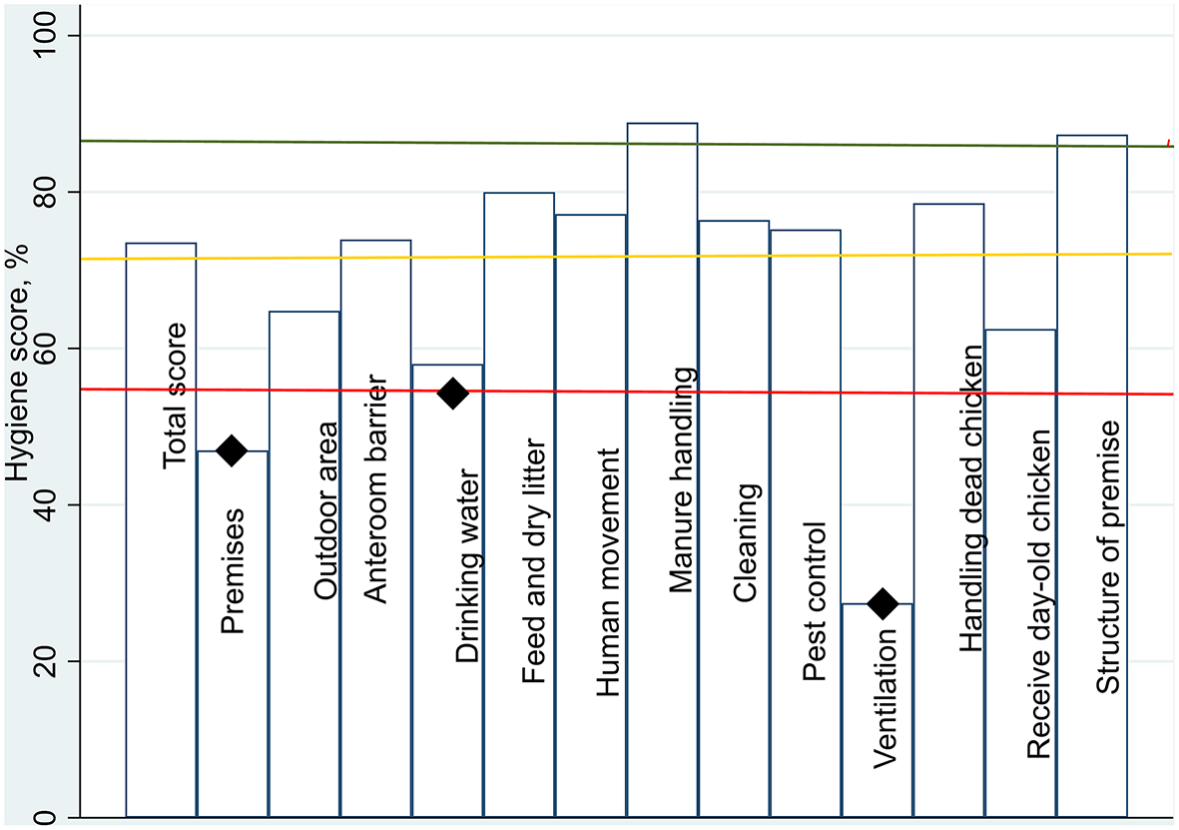
Example of a graph summary from one farms’ hygiene results. The farms’ total score is the bar to the left, and the horizontal lines indicate the assessment limits for hygiene rating. Below 55% (red line) indicates poor hygiene, between 55.1–70% (yellow line) indicates acceptable hygiene and above 85% (green line) indicates excellent hygiene. The diamonds point out the biosecurity areas needing improvement with scores below or borderline to 55% according to assessment scores

The results from the HPR-F protocol were presented to the individual farmers, with a summary of the 13 categories and improvement areas pointed out for each farm. Figure 1 shows the results from one selected farm. The bars for each category (in %) show the hygienic results according to the three vertical lines indicating poor hygiene, acceptable hygiene and excellent hygiene.

### Hygiene performance results by campylobacter-status

**Fig. 2:**
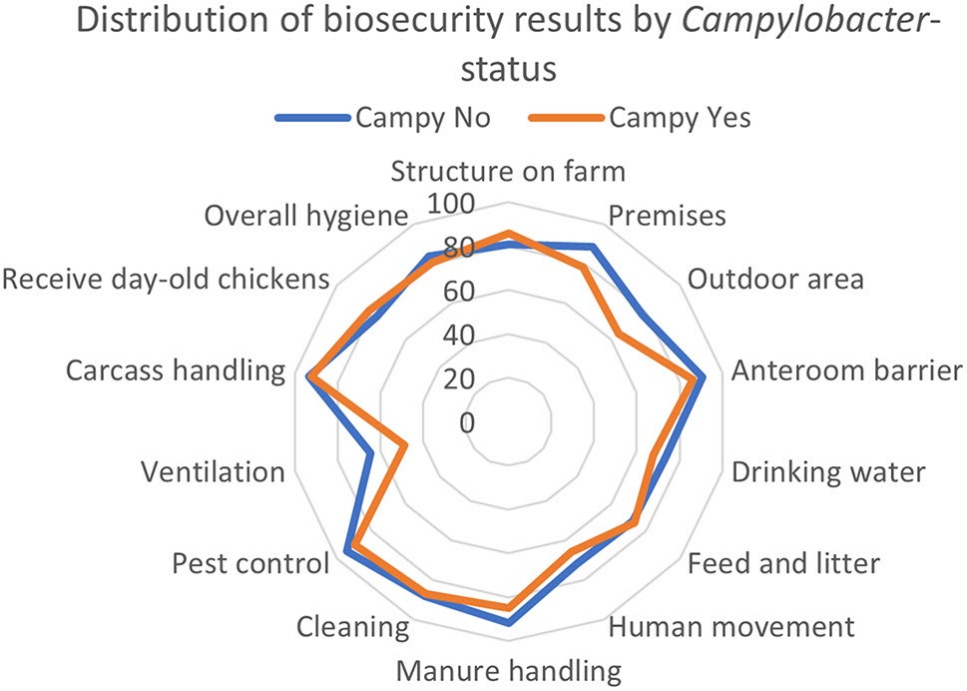
Distribution of mean results (%) in the HPR-F protocol according to *Campylobacter*-status. Blue line indicating no *Campylobacter*-cases and orange line indicating one, two or three *Campylobacter*-cases in years 2019–2021

For the farms included in the study, 13 farms were categorised as controls (no *Campylobacter*-colonisations in three years) and 17 farms as cases (one, two or three *Campylobacter*-colonisations in three years) during 2019–2021. The biosecurity results by *Campylobacter*-status are presented in Fig. 2. The mean overall hygiene score was 80.5% and 83.6% for the *Campylobacter*-cases and -controls, respectively. Logistic regression analyses were performed with Outdoor area, Human movement, Manure handling and Ventilation as explanatory variables and *Campylobacter*-status (0,1) as response variable. The multivariable logistic model was unstable, most likely due to limited n. The variable Outdoor Area was the most stable univariable factor. The questions in this category was regarding concrete/solid area around premises, rainwater transported away from building, anteroom-barrier for entrance of visitors, intake of equipment, two gates in the building, vegetation surrounding premises and feed spilling. A final model was established using the sum of scores of Outdoor area, Human movement, Manure handling and Ventilation as predictor. This model was more stable and indicated a linear relationship between the sumscore and *Campylobacter*-status with Odds ratio = 0.95, 95% Confidence interval = 0.92–0.99 and *P* = 0.007. Assessed by correlation, the four predictors showed limited correlation, indicating measuring various aspects of biosecurity. The multivariable model without sum of scores, and instead each predictor variable included in the model, was abandoned due to limited n and little variation in the dataset on hygiene score in the different categories. Thus, it was not possible to statistically predict any significant association between *Campylobacter*-status and individual categories of biosecurity.

## Discussion

This study showed that the hygienic standard of the 30 investigated farms was evaluated as being on average good where 97% of the farms had “excellent” or “good” hygiene according to previous hygiene assessment (Table 1). The HPR-F total score mean was 81.9% for all 30 farms ranging from a minimum of 69.2 to a maximum of 92.2%. In this study no farms had poor hygiene routines, on the contrary, all farms had medium to high biosecurity standards and the variation in the study population was relatively small. This is a promising finding, but still, the need to identify causes for encountering *Campylobacter*-colonisation in the broiler flocks remains. HPR-F present a strength in this matter, as the questions and control points are detailed and systematic, and reflect all parts of the broiler production. To maintain a high level of biosecurity and control of hygiene, a continuous effort must be conducted that demands full attention and compliance with the set of hygiene rules. As a comparison, the overall total biosecurity score in European broiler farms was 70.9% in the study from 5 EU countries using the risk-based weighted scoring system Biocheck.Ugent [2].

The analysis of data from our study showed that poorer hygiene scores for Outdoor area, Human movement (personnel in and out of the premises), Manure handling and Ventilation were areas associated with having *Campylobacter*-colonisations in the period 2019–2021. The questions included in the category Manure handling were if the chicken house was emptied for manure directly after emptying the house for chickens, if the manure was stored without risk of contamination or spilling to chicken house or drinking water source, if remnants of manure were completely removed from area around chicken house immediately after emptying the house of manure, if there was spreading of manure of animal origin around the chicken house or on neighbouring farms and if premises were completely emptied for manure before cleaning started. These questions could reveal if there were breaches in the hygienic routines regarding manure handling. For Ventilation, the questions were if all air-intake was through intended intakes, which type of ventilation valves there were in the house, if on warm days there would be a need for more ventilation through open windows and doors, if there were fly nets on all air intakes, if there were gable fans or large valves where air inflow happens and if there were covers on the gable fans to hinder insects from entering when fans are off. Again, these detailed questions can contribute with important information in securing biosecurity by improving ventilation systems. The only question in the Outdoor area category that showed significant differences between case-control groups, was having two gates instead of one into the broiler premises.

Other hygiene factors were borderline associated with *Campylobacter*-status. With a larger study population or a larger hygienic variation between the farms, these factors may have been significantly associated with the *Campylobacter*-status.

HPR-F represents a valuable tool in the continuous work for improving the biosecurity on broiler farms. The difference in total hygiene score between farms having *Campylobacter*-cases for the three years (2019–2021) and those that didn’t, was not significant. Risk factors that can hamper biosecurity have been considered to play an important role in the introduction and spread of animal diseases in different species, and previous studies showed that biosecurity level was significantly lower in infected herds compared to uninfected herds [18]. Further, the biosecurity practices and importance of farmer’s knowledge, attitude and personality traits are previously studied [19]. By repeating the HPR-F in farms several times and over time, the biosecurity level can be monitored and different farms can be compared. Such a mapping of biosecurity can be an important tool in investigating epidemic disease outbreaks.

The results from this limited testing of HPR-F in one geographic area cannot be extrapolated to the remaining broiler farms in Norway but with an extended HPR-F registration of a large number of farms, the mapping of high and low biosecurity farms can be achieved in the future.

The introduction of *Campylobacter* into a broiler flock can go by many different routes, and there is very often no obvious cause of introduction and colonization. It is therefore important to have a systematic and in-depth system in place which can detect failure to comply with the biosecurity system in broiler farms. Biosecurity on broiler farms can be enhanced and this could contribute to a reduction in flock colonization [19]. But we are very aware that *Campylobacter*-cases sporadically occur. In Norway, no distinct “repeaters” exist when it comes to *Campylobacter*-positive farms. Over a period of several years there are very few farms that have *Campylobacter*-positive flocks repeatedly. The needle in the haystack is almost always difficult to find, but we believe that the HPR-F protocol is a good tool to work systematically and to address faults in a pedagogic way to the farmers.

The Biocheck.UGent [13], a quantitative tool to measure biosecurity, includes an evaluation of risk for each biosecurity measure and this is also incorporated in HPR-F as the hygienic weighing for each question in the 13 categories. The hygienic and economic weighing gives important information to the farmer about the most important areas to work with in order to obtain improved biosecurity on the farm.

Six assessors conducted the HPR-F, and they were all trained before doing the registrations. There were no significant differences between the assessor’s registrations in HPR-F and this adresses the importance of pre-training personell when doing the audit.

According to the The European Union One Health 2022 Zoonoses Report [20], 18.1% of sampled units of broilers in EU-member states were *Campylobacter*-positive and 10.5% were positive in non-EU member states. The flock prevalence of *Campylobacter* in broilers according to the Norwegian Action Plan against *Campylobacter* was 4.8–6.1% for the years 2020–2023 [5]. This is thus lower compared to most other European countries, and in addition broiler meat from positive flocks is either heat treated or deep-frozen (-18 °C) for 3 weeks before being presented to market [5]. The cost of this treatment is high and demands resources from farmers, abattoirs and food safety authorities. A reduction in the number of *Campylobacter*-positive flocks would contribute to cost reduction and therefore the effort to achieve the highest possible biosecurity level in broiler production is an important task. In addition, the handling of detrimental animal diseases such as avian influenza and Newcastle disease in broiler production, requires an even higher guard and continuous improvement of biosecurity. HPR-F can be a valuable tool in the systematic evaluation of biosecurity in broiler farms, and can also be adjusted to other animal productions.

HPR-F can be conducted during different periods or chapters of production including (1) empty house facilities before new chickens arrive, (2) initial time of broilers in the production unit, (3) feeding/rearing period and (4) plucking/emptying the production facilities. In the present study, the time of registration was not included in the analysis of data due to the restricted study time available. Repeated registrations in HPR-F in different periods of the production cycle will increase the information on biosecurity level throughout the production period. In addition, the individual farmer can make comparisons to the previous registrations to look for areas of improvement. Since HFR-F is an audit scheme, the importance of doing good observations of farmers’ actual practice is crucial. This is time-consuming and the observation of everyday practice is not caught by the HPR-F protocol. Repeated registration throughout the production period can help to increase the amount of information. In addition, the questions for each of the 13 categories included in the HPR-F spreadsheet can be relevant for several chapters or production periods, hence a crosstabulation occurs with the same questions being relevant for activities in different chapters.

A UK study described the attitudes and perceptions of broiler farmers on biosecurity measures for controlling *Campylobacter* and concluded that farmers were aware of their responsibility to comply with rules [21]. Even so, the study reported frustration and uncertainty among broiler farmers on how important their biosecurity measures were in protecting their animals from *Campylobacter*-colonisation. Communication with and education of farmers on biosecurity measures are crucial components to succeed in reducing, or removing, *Campylobacter* in broiler farms. HPR-F is in addition to being an audit tool also a didactic tool for educating farmers on biosecurity. The individual farmers can follow their development on hygiene score and due to the level of detail in questions in HPR-F, the identification of concrete areas to improve is facilitated. The farmer can analyse the farm report to further identify the specific weaknesses in the 170 observations divided into the 13 categories. The next audits will then identify trends over a period of time and improvements or changes for specific areas, and should be a useful tool for hygiene improvements for the farmers.

## Conclusions

HPR-F is an innovative tool that allows the study of biosecurity measures in a quantitative manner. In this limited material we were not able to find obvious associations between low hygiene score and *Campylobacter*-positive flock or vice versa; having high hygiene score and *Campylobacter*-negative flock. That being said, we believe that the HPR-F protocol is a useful tool for detailed screening of a farm or a production for pointing out areas to improve. This will result in more robust production, that is able to withstand the introduction of pathogens.

## Data Availability

The datasets used and/or analyzed during the current study are available from the corresponding author on reasonable request.

## Declarations

### Prior publication

Data have not been published previously.
